# Digital health device measured sleep duration and ideal cardiovascular health: an observational study

**DOI:** 10.1186/s12872-021-02284-z

**Published:** 2021-10-14

**Authors:** Jane A. Leopold, Elliott M. Antman

**Affiliations:** 1 Division of Cardiovascular Medicine, Brigham and Women’s Hospital, Harvard Medical School, 77 Avenue Louis Pasteur, NRB0630K, Boston, MA 02115 USA; 2 Division of Cardiovascular Medicine, Brigham and Women’s Hospital, 350 Longwood Ave., Boston, MA 02115 USA

**Keywords:** Sleep, Phenogroups, Digital health devices, Surveys, Ideal cardiovascular health

## Abstract

**Background:**

Studies relying on self-reported sleep data suggest that there is an association between short and long sleep duration and less than ideal cardiovascular health. Evidence regarding the feasibility of using digital health devices to measure sleep duration and assess its relationship to ideal cardiovascular health are lacking. The objective of the present study was to utilize digital health devices to record sleep duration and examine the relationship between sleep duration and ideal cardiovascular health.

**Methods:**

A total of 307 participants transmitted sleep duration data from digital health devices and answered the Life’s Simple 7 survey instrument to assess ideal cardiovascular health. Sleep duration was defined as adequate (7 to < 9 h per night) or non-adequate (< 7 h and ≥ 9 h).

**Results:**

We identified three sleep-cardiovascular health phenogroups: resilient (non-adequate sleep and ideal cardiovascular health), uncoupled (adequate sleep and non-ideal cardiovascular health) or concordant (sleep and cardiovascular health metrics were aligned). Participants in the resilient phenogroup (n = 83) had better cardiovascular health factor profiles (blood pressure, blood glucose and cholesterol levels) and behaviors (healthy weight, diet, exercise, smoking) than participants in the concordant (n = 171) and uncoupled (n = 53) phenogroups. This was associated with higher Life’s Simple 7 Health Scores in the resilient phenogroup compared to the concordant and uncoupled phenogroups (7.8 ± 0.8 vs. 7.0 ± 1.4 vs. 5.6 ± 0.7, *P* < 0.01).

**Conclusion:**

This study identified three distinct sleep-ideal cardiovascular health phenogroups and highlights the advantage of incorporating sleep assessments into studies of cardiovascular health. Future studies should focus on the relationship between sleep-cardiovascular phenogroups and clinical outcomes.

*Clinical Trial Registration* Clinicaltrials.gov NCT02958098. Date of registration: November 11, 2016.

## Background

Accumulating evidence demonstrates the relationship between sleep and cardiovascular disease. Recent estimates from the United States indicate that only 65.2% of individuals self-report a sleep duration of 7 to < 9 h per night as recommended by the American Academy of Sleep Medicine [1, 2]. The relationship between sleep duration and cardiovascular disease has been described as a U-shaped association [3]. Studies have found that individuals that self-report very short (< 6 h) or long (≥ 9 h) sleep duration have an increased incidence and prevalence of hypertension, coronary heart disease, and cerebrovascular disease [4–10]. The relationship between short sleep duration and cardiovascular diseases has been attributed to surges in blood pressure, sympathoexcitatory effects, cardiac conduction system abnormalities, vascular dysfunction, impaired glucose homeostasis, inflammation, and circadian rhythm disruption (reviewed in [4]). In contrast, the mechanisms underlying the adverse effects of long sleep duration on the development of cardiovascular diseases are less well characterized. It has been suggested that long sleep duration is a marker for other health factors and behaviors, such as metabolic syndrome, tobacco use, or low levels of physical activity [4].

In 2010, the American Heart Association developed the Life’s Simple 7 survey to assess ideal cardiovascular health [11]. The survey instrument defines ideal cardiovascular health as a composite of 7 modifiable health factors and behaviors, including healthy weight, blood pressure, cholesterol, and blood glucose as well as a healthy diet, absence of tobacco use, and moderate and vigorous exercise [11, 12]. The overall Life’s Simple 7 Health Score has been linked to cardiovascular disease with lower scores associated with an increased incidence of adverse cardiovascular outcomes: for each 1 unit decrease in the score, there is an associated 12% increased risk of adverse cardiovascular events [13–15]. Similarly, individuals that have ideal scores for fewer than five of the 7 categories evaluated by the Life’s Simple 7 survey have an increased risk for developing cardiovascular disease [16, 17].

Recently, investigators have begun to examine the relationship between sleep duration and ideal cardiovascular health. One study that included only women found that women who self-reported adequate sleep duration, defined as ≥ 7 h per night, were more likely to have ideal scores in five or more Life’s Simple 7 health factors and habits categories [18]. Another cross-sectional study performed using National Health and Nutrition Examination Survey (NHANES) data found that individuals who self-reported a very short (< 6 h) or a very long (≥ 9 h) sleep duration had decreased odds of ideal cardiovascular health [19]. In the current study, we hypothesized that digital health devices would provide an objective measurement of sleep duration and allow us to evaluate the relationship between sleep duration and ideal cardiovascular health.

## Methods

### Study cohort

The My Research Legacy study was a direct-to-participant study sponsored by the American Heart Association (NCT 02958098) and open for enrollment between November 2016 and October 2018 [20]. The study was approved by the Advarra Institutional Review Board (#31995) and all participants signed informed consent. The study was open to participants in the United States who were ≥ 18 years old and had internet access. Participants self-reported baseline demographics, their history of cardiovascular disease, and completed the Life’s Simple 7 survey instrument [11, 12]. A subset of participants were provided with a Fitbit Charge 2 device, which incorporates sleep staging [21], or registered their own digital health device with the study. Participants that had digital scales linked these to their digital health device. Participant digital health data was uploaded to Validic (Validic Inc., Durham, NC). Deidentified data were parsed into sleep, weight, fitness, and routine categories via the Validic API, and transmitted to secure severs managed by The Broad Institute (Cambridge, MA) and REAN Cloud LLC (Herndon, VA).

### Sleep duration and ideal cardiovascular health

Sleep duration was determined by obtaining an average of total sleep hours recorded from 7 consecutive days to account for daily and workday-weekend variations. When 7 consecutive days were not available, an average of the consecutive days available was used. Adequate sleep duration was defined as ≥ 7 to < 9 h per night based on recommendations from the American Academy of Sleep Medicine and non-adequate sleep duration was defined as < 7 h and ≥ 9 h per night [1]. Light, deep, and rapid eye movement (REM) sleep stages were determined using post-processed heart rate signals by proprietary algorithms specific to the device manufacturer. Sleep stage duration was obtained by averaging data obtained over 7 days.

Ideal cardiovascular health was assessed using the Life’s Simple 7 survey tool. Participants answered questions related to health factors (weight, blood pressure, cholesterol and blood glucose levels) as well health behaviors (diet, exercise, smoking). The Life’s Simple 7 survey provides an assessment of ideal cardiovascular health by assigning participants a score of 0, 1, or 2 (poor, intermediate, and ideal) for 7 cardiovascular health and habit categories according to criteria defined by a panel of experts. A final Health Score, which takes into consideration medication use to manage cardiovascular risk factors is calculated and can range between 0 (poor) and 10 (ideal) [11, 12]. Ideal cardiovascular health was defined by an overall Health Score of > 7.0 or by meeting ideal criteria for a minimum of 5 categories [22, 23].

### Statistical analysis

Sample size to assess sleep data was calculated based on a reported prevalence of non-adequate sleep ranging from 26.3% to 46.3% in the general population [24]. We assumed that our study population would have a similar prevalence of non-adequate sleep. To achieve 90% power with an alpha = 0.05, the minimum sample size required was 254 participants.

Data are presented as mean ± SD and *P* values < 0.05 were considered significant. Comparisons between continuous variables were analyzed using t-tests or one-way ANOVA as applicable. Post-hoc pairwise comparisons between groups was performed using Bonferroni correction testing. Comparisons between categorical variables were analyzed using the chi-square test or Fisher’s exact test. Nonparametric testing was done using the Wilcoxon-rank sum test or Kruskal–Wallis test. Post-hoc pairwise comparisons between groups was performed using Dunn’s test. Data were analyzed using Stata 15/SE 15.1 (StataCorp LLC, College Station, TX) and Prism 9.0 (GraphPad, San Diego, CA).

## Results

**Fig. 1:**
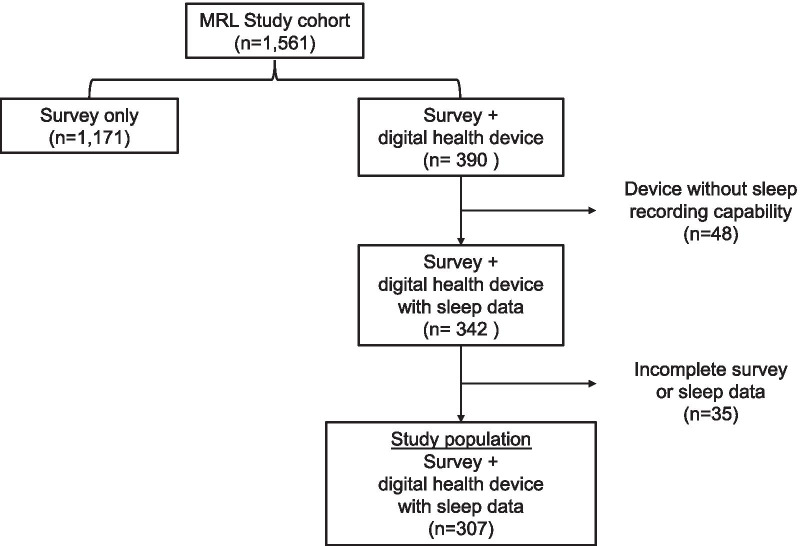
Study Flowchart

A total of 342 participants contributed sleep data from their registered digital health devices. From this group, 35 participants were excluded due to incomplete Life’s Simple 7 data or incomplete recorded sleep data leaving a final sample size of 307 participants (Fig. 1). There were 146 participants who were categorized as having adequate sleep duration (≥ 7 to < 9 h/night) and slept an average of 7.6 ± 0.5 h/night. The remaining 161 participants who were categorized as having non-adequate sleep (< 7 [n = 144] and ≥ 9 [n = 17] hours/night) slept an average of 6.4 ± 1.2 h/night. A subset of participants (n = 131 with non-adequate sleep and n = 84 with adequate sleep) provided sleep stage data. Compared to participants with non-adequate sleep, individuals with adequate sleep spent more time in deep sleep (58.1 ± 17.2 vs. 69.1 ± 16.8 min/night, *P* < 0.01) and REM sleep (75.6 ± 22.6 vs. 97.0 ± 20.6 min/night, *P* < 0.01). Participants with non-adequate sleep were less likely to have ideal cardiovascular health when assessed by having an ideal score in 5 or more Life’s Simple 7 health factors and behaviors categories (21.7% vs. 37.0%, *P* < 0.01) or by the Life’s Simple 7 Health Score (6.8 ± 1.3 vs. 7.2 ± 1.4, *P* < 0.01).

**Fig. 2:**
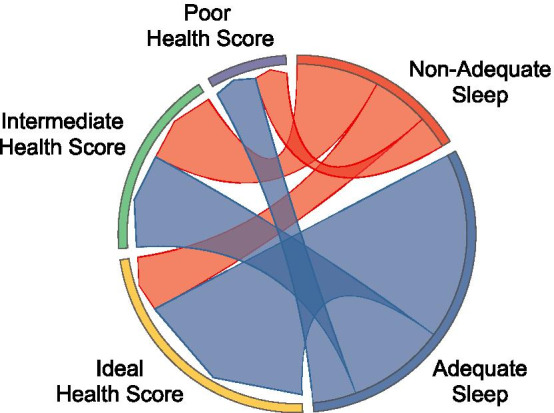
Relationship between sleep and cardiovascular health score to define phenogroups. Sleep duration was defined as adequate (≥ 7 to < 9 h) or non-adequate (< 7 and ≥ 9 h). Cardiovascular health was defined as ideal (Life’s Simple 7 Health Score: > 7), intermediate (Life’s Simple 7 Health Score: > 5 to ≤ 7), or poor (≤ 5). The relationship between sleep and cardiovascular health score revealed three phenogroups: resilient, defined by ideal cardiovascular health and non-adequate sleep (n = 83); uncoupled, defined by intermediate or poor cardiovascular health, but adequate sleep (n = 53); and concordant where sleep duration and cardiovascular health measures were aligned with adequate sleep and ideal cardiovascular health or non-adequate sleep and intermediate or poor cardiovascular health (n = 171)

Among our participants, three phenogroups that describe the sleep-ideal cardiovascular health relationship emerged: resilient, concordant, and uncoupled. The resilient phenogroup (n = 83) was characterized by ideal cardiovascular health, but non-adequate sleep while the uncoupled phenogroup (n = 53) had non-ideal cardiovascular health, but adequate sleep. The concordant phenogroup (n = 171) had alignment of cardiovascular health and sleep duration (either non-ideal cardiovascular health and non-adequate sleep or ideal cardiovascular health and adequate sleep) (Fig. 2).

**Fig. 3:**
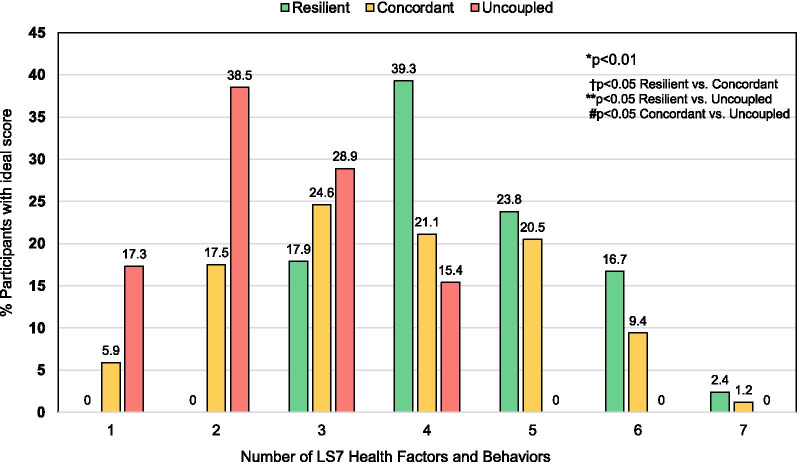
Phenogroup differences in Life’s Simple 7 health factors and behaviors scored as ideal. The 7 health factors and behaviors categories are scored as poor, intermediate, or ideal based on pre-defined criteria. The number of health factors scored as ideal for each of the phenogroups is shown. Resilient (n = 83), Concordant (n = 171), Uncoupled (n = 53). *P < 0.01 among groups by Pearson’s chi-squared test. *P* < 0.01 Resilient versus Concordant, Resilient versus Uncoupled and Concordant versus Uncoupled by post-hoc Bonferroni multiple comparisons test

**Fig. 4:**
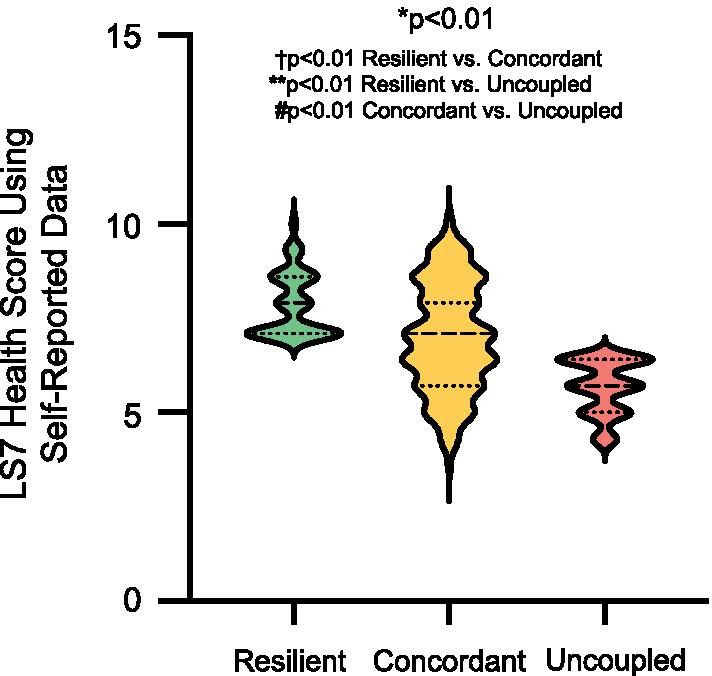
Life’s Simple 7 Health Score calculated using self-reported data. The distribution of Life’s Simple 7 Health Scores is compared between the resilient (n = 83), concordant (n = 171), and uncoupled (n = 53) phenogroups and presented as violin plots. The median and quartiles are denoted by dashed lines. *P < 0.01 among groups by Kruskal–Wallis rank test. *P* < 0.01 Resilient versus Concordant, Resilient versus Uncoupled and Concordant versus Uncoupled by Dunn’s post hoc test

Participants in the resilient phenogroup were younger (41.2 ± 13.7 vs. 43.2 ± 13.2 vs. 47.1 ± 10.8 yrs, *P* < 0.04) than those in the concordant and uncoupled phenogroups and were less likely to have a history of diabetes mellitus (*P* < 0.01), hypertension (*P* < 0.05), or hypercholesterolemia (*P* < 0.01) or to take medications for these cardiovascular disease risk factors (all *P* < 0.01) (Table 1). Participants in the resilient group also had significantly lower weight and body mass index (25.2 ± 4.2 vs. 28.4 ± 7.1 vs. 33.0 ± 6.0 kg/m^2^, *P* < 0.01) than the concordant and uncoupled phenogroups as well as lower systolic blood pressures (*P* < 0.04) and blood glucose levels (*P* < 0.03) (Table 1). There were also differences between the groups with respect to other modifiable cardiovascular health behaviors, such as diet and exercise. Individuals in the resilient phenogroup consumed more daily servings of vegetables (*P* < 0.01), fruits (*P* < 0.03), and whole grains (*P* < 0.01) than individuals in the uncoupled phenogroup. While there was no difference between the phenogroups with respect to minutes of moderate exercise per week, both the resilient and concordant phenogroups self-reported more minutes of vigorous exercise per week than individuals in the uncoupled phenogroup (76.5 ± 115.6 vs. 98.2 ± 127.1 vs. 34.8 ± 64.1 min/week, *P* < 0.01). Participants in the resilient phenogroup were more likely to have ideal scores in 5 or more Life’s Simple 7 health factors and behaviors (Fig. 3). Participants in the resilient phenogroup also had better Life’s Simple 7 Health Scores than participants in the concordant or uncoupled phenogroups (7.8 ± 0.8 vs. 7.0 ± 1.4 vs. 5.6 ± 0.7, *P* < 0.01)(Fig. 4).

**Table 1:** Self-reported demographics, risk factors, health factors, diet and exercise

	Resilient (n = 83)	Concordant (n = 171)	Uncoupled (n = 53)	*P* value
*Demographics*				
Age (yrs)^b^	41.2 ± 13.7	43.2 ± 13.2	47.1 ± 10.8	< 0.04
Female (%)	88.0	77.8	79.3	0.15
Race and ethnicity (no.)				0.87
Asian	3	6	0	
Black	3	6	1	
Hispanic	3	9	2	
White	71	146	47	
Other	3	4	3	
Region (no.)				0.96
Northeast	14	26	11	
South	31	66	18	
Midwest	24	47	14	
West	14	33	10	
Diagnosed with cardiovascular disease (%)	41.0	36.8	45.3	0.52
Diabetes mellitus (%)^b,c^	0	5.8	15.1	< 0.01
Hypertension (%)^b^	38.6	46.2	60.4	< 0.05
Hypercholesterolemia (%)^b,c^	38.6	49.7	75.5	< 0.01
Medications (%)				
Diabetes mellitus^b,c^	0	4.1	15.1	< 0.01
Hypertension^a,^^b,c^	10.8	25.1	45.3	< 0.01
Hypercholesterolemia^b^	10.8	18.7	32.1	< 0.01
Smoking status (%)				0.07
Current	0	1.2	1.9	
Quit < 12 months	1.2	2.3	5.7	
Quit ≥ 12 months	21.7	23.4	39.6	
Never	77.1	73.1	52.8	
*Clinical data*				
Weight (kg)^a,^^b,c^	71.2 ± 13.8	80.4 ± 20.1	93.2 ± 19.0	< 0.01
BMI (kg/m^2^)^a,^^b,c^	25.2 ± 4.2	28.4 ± 7.1	33.0 ± 6.0	< 0.01
Systolic blood pressure (mmHg)^a,d^	114.1 ± 10.3	118.3 ± 13.4	117.9 ± 10.9	< 0.04
Diastolic blood pressure (mmHg)^d^	71.4 ± 7.3	73.0 ± 8.5	73.1 ± 7.5	0.28
Total cholesterol (mg/dL)^d^	183.1 ± 29.6	188.0 ± 31.4	194.2 ± 27.8	0.11
Blood glucose (mg/dL)^b,d^	94.6 ± 8.0	98.1 ± 18.1	101.8 ± 15.7	< 0.03
*Diet*				
Vegetables/day (cups)^b^	2.2 ± 1.5	1.8 ± 1.3	1.5 ± 0.9	< 0.01
Fruit/day (cups)^b^	1.6 ± 1.1	1.3 ± 1.0	1.2 ± 0.8	< 0.03
Fish (servings/week)	1.0 ± 1.2	0.9 ± 1.0	1.0 ± 1.2	0.66
Whole grains (servings/day)^b,d^	1.8 ± 1.4	1.8 ± 1.3	1.2 ± 0.8	< 0.01
Sugar-sweetened beverages (servings/week)	1.7 ± 2.9	1.6 ± 2.4	2.1 ± 2.8	0.56
Avoid prepackaged foods (%)	54.2	50.9	39.6	0.23
Avoid eating out (%)	39.8	36.3	26.4	0.27
Avoid salt at home (%)	61.5	60.2	54.7	0.72
*Exercise*				
Moderate exercise (min/week)	198.7 ± 168.3	218.9 ± 223.9	172.4 ± 161.0	0.32
Vigorous exercise (min/week)^c^	77.5 ± 115.6	98.2 ± 127.1	34.8 ± 64.1	< 0.01
*LS7 score*				
Smoking score (%)^b^				0.82
Poor	0	1.2	1.9	
Intermediate	1.2	2.3	5.7	
Ideal	98.8	96.5	92.5	
Activity score (%)^b,c^				< 0.02
Poor	0	1.2	3.8	
Intermediate	26.5	32.2	45.3	
Ideal	73.5	66.6	50.9	
Diet score (%)^b,c^				< 0.01
Poor	31.3	39.8	62.3	
Intermediate	54.2	50.9	35.9	
Ideal	14.5	9.3	1.8	
Weight score (%)^a,^^b,c^				< 0.01
Poor	13.3	33.9	66.0	
Intermediate	32.5	29.8	30.2	
Ideal	54.2	32.3	3.8	
Blood glucose score (%)^b,c^				< 0.02
Poor	0	4.1	3.8	
Intermediate	22.9	28.7	49.1	
Ideal	77.1	67.2	47.1	
Cholesterol score (%)^a,^^b,c^				< 0.01
Poor	1.2	2.3	1.9	
Intermediate	32.5	48.0	73.6	
Ideal	66.3	49.7	24.5	
Blood pressure score (%)^a,^^b^				< 0.01
Poor	1.2	5.9	0	
Intermediate	37.4	53.8	75.5	
Ideal	61.4	40.3	24.5	
Health score^a,^^b,c^	7.8 ± 0.8	7.0 ± 1.4	5.6 ± 0.7	< 0.01

Categorical variables are analyzed by Chi-Square test. Continuous variables are analyzed by ANOVA. Post-hoc comparisons were done by Bonferroni correction. Non-parametric variables were analyzed by Kruskal–Wallis test. Post-hoc comparisons were done by Dunn’s test

^a^*P* < 0.05 Resilient versus Concordant

^b^*P* < 0.05 Resilient versus Uncoupled

^c^*P* < 0.05 Concordant versus Uncoupled

^d^Contains imputed data from Life’s Simple 7

We next sought to explore the concordant phenogroup in more detail as this group was comprised of participants who had adequate sleep and ideal cardiovascular health and were considered to have a healthy phenotype (n = 104) as well as those individuals with inadequate sleep and non-ideal cardiovascular health who were considered to have an unhealthy phenotype (n = 67). Those individuals with a healthy phenotype were younger (40.3 ± 13.5 vs. 47.6 ± 11.4 yrs, *P* < 0.01), less likely to have hypertension (34.6 vs. 64.2%, *P* < 0.01), diabetes mellitus (1.9 vs. 11.9%, *P* < 0.01), or hypercholesterolemia (35.6 vs. 71.6%, *P* < 0.01), and had a lower body mass index (25.9 ± 5.2 vs. 32.2 ± 7.9 kg/m^2^, *P* < 0.01) than unhealthy individuals. Individuals in the healthy phenotype group also subscribed to a heart healthy diet with a greater intake of daily vegetables (2.1 ± 1.3 vs. 1.4 ± 1.0 cups/day, *P* < 0.01), fruits (1.5 ± 1.0 vs. 1.0 ± 0.7 cups/day, *P* < 0.01), servings of fish/week (1.0 ± 1.1 vs. 0.7 ± 0.8 servings/week, *P* < 0.05), and fewer servings of sugar-sweetened beverages per week (1.1 ± 1.9 vs. 2.3 ± 3.0 cups/day, *P* < 0.01). While there was no difference between the two groups with respect to weekly minutes of moderate exercise, the healthy group performed more weekly minutes of vigorous exercise (126.6 ± 134.8 vs. 54.2 ± 100.2 min, *P* < 0.01) than the unhealthy group.

The digital health devices used to measure sleep duration also measured weight, activity, and daily step count. Weight measured by digital health devices and calculated body mass index remained lower in participants in the resilient phenogroup (n = 75) as compared to the concordant (n = 145) and uncoupled (n = 47) phenogroups (both *P* < 0.01) (Table 2). Participants in the resilient phenogroup also tended to underreport their weight while individuals in the uncoupled phenogroup overreported their weight on the Life’s Simple 7 survey as compared to what was measured by the digital health device (*P* < 0.01). Using digital health device measured weight, there was a significant difference in the distribution of participants with poor, intermediate and ideal scores between the phenogroups (*P* < 0.01). Interestingly, using digital health device measured exercise data, participants in the resilient phenogroup performed fewer minutes per week of moderate exercise than the other phenogroups (*P* < 0.04), but similar minutes per week of vigorous exercise. This resulted in a similar distribution of participants with intermediate and ideal activity scores among the phenogroups. Participants in the three phenogroups also recorded a similar number of daily steps, suggesting that contact time with their digital health devices was similar between the groups.

**Table 2:** Digital health device data

	Resilient (n = 75)	Concordant (n = 145)	Uncoupled (n = 47)	*P* value
Weight (kg)^a,b,c^	71.7 ± 14.6	81.7 ± 21.4	92.4 ± 19.5	< 0.01
Difference between self-reported and digital device measured (kg)^b,c^	−1.1 ± 3.7	−0.9 ± 4.1	1.3 ± 5.5	< 0.01
BMI (kg/m^2^)^a,b,c^	25.5 ± 4.5	28.9 ± 7.5	32.6 ± 6.3	< 0.01
Difference between self-reported and digital device measured (kg/m^2^)^b,c^	-0.4 ± 1.3	-0.3 ± 1.5	0.4 ± 1.9	< 0.01
Healthy weight score (%)^a,b,c^				< 0.01
Poor	16.0	35.2	61.7	
Intermediate	32.0	31.0	34.0	
Ideal	52.0	33.8	4.3	
LS7 Health Score with Device Weight Score^a,b,c^	7.8 ± 0.8	7.0 ± 1.4	5.8 ± 0.6	< 0.01

	Resilient (n = 77)	Concordant (n = 164)	Uncoupled (n = 51)	*P* value
Moderate exercise (min/week)^a^	99.6 ± 116.6	158.6 ± 188.1	120.4 ± 174.9	< 0.04
Difference between self-reported and digital device measured (min)	102.1 ± 202.6	66.4 ± 254.0	54.3 ± 219.4	0.45
Vigorous exercise (min/week)	160.3 ± 187.2	199.5 ± 284.4	133.5 ± 153.8	0.18
Difference between self-reported and digital device measured (min)	−77.9 ± 179.9	−97.0 ± 282.1	−97.9 ± 156.0	0.83
Physical activity score (%)				0.56
Poor	0.0	0.0	0.0	
Intermediate	37.7	36.0	47.1	
Ideal	62.3	64.0	52.9	
LS7 health score with device activity score^a,b,c^	7.8 ± 0.9	7.0 ± 1.4	5.8 ± 0.7	< 0.01

	Resilient (n = 71)	Concordant (n = 139)	Uncoupled (n = 45)	*P* value
LS7 Health score with both weight and activity score^a,b,c^	7.7 ± 0.9	7.0 ± 1.5	5.9 ± 0.7	< 0.01

	Resilient (n = 84)	Concordant (n = 171)	Uncoupled (n = 52)	*P* value
Daily steps	8027.4 ± 4020.4	8794.5 ± 5083.5	7683.0 ± 3948.8	0.22

Categorical variables are analyzed by Chi-Square test. Continuous variables are analyzed by ANOVA. Post-hoc comparisons were done by Bonferroni correction. Non-parametric variables were analyzed by Kruskal–Wallis test. Post-hoc comparisons were done by Dunn’s test.

^a^*P* < 0.05 Resilient versus Concordant

^b^*P* < 0.05 Resilient versus Uncoupled

^c^*P* < 0.05 Concordant versus Uncoupled

## Discussion

In the current study, we used digital health device recorded sleep data and the validated American Heart Association’s Life Simple 7 survey tool to identify sleep-ideal cardiovascular health phenogroups. Using American Academy of Sleep Medicine guidelines to define adequate and non-adequate sleep, we found that participants segregated into one of three phenogroups: resilient, uncoupled, or concordant. The resilient, uncoupled, and concordant phenogroups are based on having ideal cardiovascular health, but non-adequate sleep; non-ideal cardiovascular health and adequate sleep; or matched cardiovascular health and sleep status, respectively. When examining differences between these phenogroups, we found that individuals belonging to the resilient phenogroup were younger and had a lower prevalence of diabetes mellitus, hypertension, and hypercholesterolemia than the other groups while participants in the uncoupled group had a high burden of cardiovascular risk factors. There were also significant differences between the phenogroups with respect to modifiable health behaviors, such as diet and exercise. When digital device recorded weight was examined, differences between the phenogroups persisted. Interestingly, digital device recorded exercise data revealed that individuals in the concordant and uncoupled groups performed more minutes per week of vigorous exercise than they reported. Taken together, these findings demonstrate that acquiring digital health device recorded sleep duration is not only feasible in studies of ideal cardiovascular health but is likely to be more reliable than survey data. Furthermore, digital health device recorded sleep duration should be included in these types of studies as it revealed that the relationship between sleep duration and ideal cardiovascular health is more complex than previously reported.

Although prior studies have examined the relationship between ideal cardiovascular health and sleep duration, these studies relied on self-reported sleep duration and did not identify or describe the resilient, concordant, and uncoupled phenogroups. One large study of 7784 individuals without cardiovascular disease who participated in the NHANES study reported that participants with very short (< 6 h) or very long (≥ 9 h) sleep duration had decreased odds of ideal cardiovascular health. Among study participants, only 21.3% had ideal cardiovascular health defined as having an ideal score for a minimum of five of 7 cardiovascular health factors and behaviors evaluated by the Life’s Simple 7 survey [19]. In contrast to the NHANES study, our study measured sleep duration via digital health device, included participants with prevalent cardiovascular disease, and defined ideal cardiovascular health by the calculated Life’s Simple 7 Health Score [11, 22]. Furthermore, we found that only a subgroup of individuals who had short or very long sleep duration had non-ideal cardiovascular health scores. Another study that examined the relationship between self-reported sleep habits and ideal cardiovascular health in women reported that adequate sleep duration and higher quality sleep were associated with ideal scores in at least four of 7 Life’s Simple 7 cardiovascular health factors and behaviors categories [18]. Although we were not able to assess sleep quality, we also found that participants that recorded adequate sleep duration were more likely to have ideal scores in five or more Life’s Simple 7 categories.

The relationship between sleep duration and cardiovascular health is complex and sleep may be a surrogate marker for other health factors and behaviors that have adverse cardiovascular effects. A report from the United Kingdom Biobank project found that individuals who self-reported long (non-adequate) duration sleep had higher relative odds of tobacco use, lower levels of physical activity, obesity, and poor diet [25]. Other studies have reported that non-adequate sleep was associated with a higher odds ratio of impaired fasting glucose levels, diabetes mellitus, hypertension, or hypercholesterolemia [26, 27]. The importance of these relationships is demonstrated by the finding that higher levels of coronary artery calcification and higher brachial-ankle pulse wave velocity, a marker of arterial stiffness, associated with very short and very long sleep duration in a cohort of individuals without established cardiovascular disease [28]. Longitudinal studies have also reported that participants with non-adequate sleep, particularly short sleep duration, were more likely to have cardiovascular events than individuals with adequate sleep duration [29].

There are several limitations to our study that may influence generalizability of the findings. First, we enrolled a relatively small sample size and some of our participants had prevalent cardiovascular disease. Our study design was direct-to-participant and we used transmitted digital health device measured data to assess sleep duration [30]. There has been some controversy regarding the accuracy of digital health devices for recording sleep duration and stages. Several studies have evaluated the performance of digital health devices that record sleep using polysomnography and reported heterogeneous results with digital health devices overestimating, showing no difference, or underestimating standard actigraphy results. These studies, however, differed in the populations enrolled and the devices studied (reviewed in [31]), suggesting that further clinical studies are required. There are limitations related to wearable sleep trackers, including inherent differences between commercial devices, understanding device outcomes, the effects of device position on performance, and device reliability as these monitors rely on post-processed heart rate signals (reviewed in [31]). Furthermore, in our study, full sleep staging was only available in a subset of participants as some participants devices recorded in the classic as opposed to the stage mode. Since we did not administer a sleep questionnaire, we were also unable to assess sleep quality and we were unable to compare self-reported with digital health device measured sleep duration. Nonetheless, activity tracker data has been compared with patient-reported sleep outcomes and been shown to classify health status accurately in patients with heart disease [32]. Finally, we do not have longitudinal outcome data to determine if the phenogroups that we identified in our study cohort are associated with increased risk for, or protection from, cardiovascular events.

## Conclusions

In summary, our study demonstrates the utility of digital health device measured sleep duration within the context of a direct-to-participant study design for assessing the association between sleep duration and ideal cardiovascular health. Furthermore, we identified three distinct sleep-cardiovascular health phenogroups, which underscores the necessity of incorporating sleep assessments into studies of cardiovascular health and disease. These phenogroups revealed that the association between sleep duration and ideal cardiovascular health is complex and that subgroups exist where sleep duration and ideal cardiovascular health are discordant. The relationship between these phenogroups and cardiovascular outcomes should be investigated in future studies as they may have implications for therapeutic interventions to improve cardiovascular morbidity and mortality.

## Data Availability

For information regarding data availability, please contact the corresponding author. The data are not publicly available due to privacy restrictions and protection of personal data.

## Abbreviations

NHANES: National Health and Nutrition Examination Survey
REM: Rapid eye movement
